# Unique and Common Features of Innate-Like Human Vδ2^+^ γδT Cells and Mucosal-Associated Invariant T Cells

**DOI:** 10.3389/fimmu.2018.00756

**Published:** 2018-04-23

**Authors:** Nicholas M. Provine, Benedikt Binder, Michael E. B. FitzPatrick, Anita Schuch, Lucy C. Garner, Kate D. Williamson, Bonnie van Wilgenburg, Robert Thimme, Paul Klenerman, Maike Hofmann

**Affiliations:** ^1^Translational Gastroenterology Unit, Nuffield Department of Medicine, University of Oxford, Oxford, United Kingdom; ^2^Department of Internal Medicine II, University Hospital Freiburg, Freiburg, Germany; ^3^Faculty of Biology, University of Freiburg, Freiburg, Germany; ^4^Peter Medawar Building for Pathogen Research, University of Oxford, Oxford, United Kingdom

**Keywords:** mucosal-associated invariant T, γδ T cells, innate-like T cells, hepatitis C virus, mucosal immunology, Vδ2 γδ T cells

## Abstract

Mucosal-associated invariant T (MAIT) cells are innate-like T cells abundant in humans that can be activated in a TCR-independent manner by inflammatory and antiviral cytokines. In humans, the capacity for TCR-independent activation is functionally linked to a transcriptional program that can be identified by the expression of the C-type lectin receptor, CD161. In addition to MAIT cells, it has been demonstrated that a subset of γδT cells expresses CD161 and can be activated by TCR-independent cytokine stimulation. In this study, we sought to clarify the nature of cytokine-responsive human γδT cells. We could link CD161 expression on Vδ2^+^ versus Vδ1^+^ γδT cells to the observation that Vδ2^+^ γδT cells, but not Vδ1^+^ γδT cells, robustly produced IFN-γ upon stimulation with a variety of cytokine combinations. Interestingly, both CD161^+^ and CD161^−^ Vδ2^+^ γδT cells responded to these stimuli, with increased functionality within the CD161^+^ subset. This innate-like responsiveness corresponded to high expression of PLZF and IL-18Rα, analogous to MAIT cells. Vδ2^+^ γδT cells in human duodenum and liver maintained a CD161^+^ IL-18Rα^+^ phenotype and produced IFN-γ in response to IL-12 and IL-18 stimulation. In contrast to MAIT cells, we could not detect IL-17A production but observed higher steady-state expression of Granzyme B by Vδ2^+^ γδT cells. Finally, we investigated the frequency and functionality of γδT cells in the context of chronic hepatitis C virus infection, as MAIT cells are reduced in frequency in this disease. By contrast, Vδ2^+^ γδT cells were maintained in frequency and displayed unimpaired IFN-γ production in response to cytokine stimulation. In sum, human Vδ2^+^ γδT cells are a functionally distinct population of cytokine-responsive innate-like T cells that is abundant in blood and tissues with similarities to human MAIT cells.

## Introduction

The capacity of human T cells to be activated independent of TCR ligation is atypical and largely restricted to specific, defined innate-like T cell subsets, of which the mucosal-associated invariant T (MAIT) cell population is the most abundant in humans (1). A fundamental innate-like characteristic of MAIT cells is that they can be stimulated to produce IFN-γ by cytokines in the absence of exogenous TCR ligand (2). Cytokine-mediated induction of IFN-γ by T cells requires combinatorial stimulation with at least two cytokines, and a number of cytokines have been shown to be effective in this regard (3, 4). IL-12 combined with either IL-15 or IL-18 is the most potent cytokine stimulus identified. The exact *in vivo* implications of the capacity for these cells to be activated by TCR-independent stimuli remains unclear, but it has been shown to augment activation by TCR ligation and allow for the activation of MAIT cells by pathogens that do not produce the relevant TCR ligands (3, 5–7).

Intriguingly, in humans, this capacity for TCR-independent, cytokine-mediated IFN-γ production is also seen to varying degrees in conventional CD8^+^ αβT cells, CD4^+^ αβT cells, and γδT cells. Across all populations, a shared transcriptional signature is expressed by the IFN-γ-producing, cytokine-responsive subset and this signature can be identified by the expression of CD161, of which MAIT cells express the highest levels (8). While only a subset of conventional CD4^+^ and CD8^+^ αβT cells expresses CD161, a large fraction of γδT cells express CD161, and these cells respond more robustly to cytokine stimuli than conventional αβT cells. Thus, we sought to more thoroughly characterize the cytokine-responsive subset of γδT cells.

In human circulation, two major subsets of γδT cells can be identified and differentiated based on the expression of a TCR utilizing either Vδ1 or Vδ2 gene segments, hereafter Vδ1^+^ or Vδ2^+^, respectively (9). Recent work has demonstrated that the circulating Vδ1^+^ γδT cell population shares several characteristics with conventional αβT cells, with regard to high levels of clonal TCR diversity, a large pool of phenotypically naïve cells, and a small subset of clonally expanded memory cells (10). By contrast, circulating Vδ2^+^ γδT cells display many characteristics more in line with the MAIT cell population, including limited TCR sequence diversity, with up to 95% of TCRs being comprised of a Vδ2/Vγ9 pairing (11, 12). It has been demonstrated that γδT cells, including the Vδ2^+^ γδT cell subset, can be activated through a cytokine-dependent, TCR-independent stimulation process (13, 14). This is highly analogous to what has been recently reported for MAIT cells (3, 6). In total, it appears that Vδ2^+^ γδT cells share several of the innate-like T cell characteristics seen in MAIT cells.

We thus hypothesized that the previously identified CD161^+^ γδT cells and Vδ2^+^ γδT cells are in fact one and the same cell population, and represent an additional, abundant population of innate-like T cells. Consistent with this, we demonstrate that the majority of Vδ2^+^ γδT cells express CD161, thus linking the two prior reports of cytokine-responsive human γδT cells (8, 13). Extending these findings, we demonstrate that Vδ2^+^ γδT cells are present at frequencies similar to MAIT cells in liver and duodenum and maintain an innate-like phenotype and responsiveness to cytokine stimulation. However, in contrast to MAIT cells, Vδ2^+^ γδT cells did not exhibit type 17 effector functionality. Collectively, these data demonstrate that Vδ2^+^ γδT cells and MAIT cells are both abundant innate-like T cell populations that share several functional characteristics. Interestingly, we could detect preserved frequency and phenotype of Vδ2^+^ T cells in patients chronically infected with hepatitis C virus (HCV), in contrast to the known reduction in MAIT cell frequency. These data stress the importance of including studies of the Vδ2^+^ γδT cell population when investigating cytokine-mediated activation of lymphocyte populations.

## Materials and Methods

### Blood Sample Processing

Peripheral blood mononuclear cells (PBMCs) were isolated from fresh human blood by density gradient centrifugation. Briefly, blood was diluted in PBS and layered over Lymphoprep (Axis-Shield) or Pancoll (PAN Biotech). Samples were centrifuged at 973 *g* for 30 min without brake. The mononuclear cell layer was collected and washed in R10 [RPMI-1640 (Lonza) supplemented with 10% FBS (Sigma-Aldrich) and 1% penicillin/streptomycin (Sigma-Aldrich)]. Residual red blood cells were lysed by incubation in 1× ACK (Ammonium-Chloride-Potassium) lysis solution for <5 min. Cells were washed an additional time in R10 before downstream utilization or storage in liquid nitrogen for subsequent use.

### Tissue Sample Processing

Liver samples were collected from the healthy margin of patients undergoing tissue resection for metastases of colorectal cancer or hepatocellular carcinoma. Tissue was dissociated by grinding through a 70 µm filter (ThermoFisher). Duodenal samples were collected by biopsy during endoscopy for routine clinical indications. Duodenal biopsies were incubated for 1 h shaking at 37°C in a solution of R10 + 1 mg/ml Collagenase D (Sigma-Aldrich) + 100 µg/ml DNase I (ThermoFisher). Biopsies were then dissociated by vigorous agitation using a GentleMACS Dissociator (Miltenyi Biotec) and strained through a 70 µm filter. From this point, liver and duodenal samples were processed in the same way. Samples were washed once in R10 media. Mononuclear cells were isolated on a discontinuous 70–35% Percoll gradient (GE Healthcare) by centrifugation at 700 *g* for 20 min without brake. The interface containing mononuclear cells was collected and washed in R10. Residual red blood cells were lysed using 1× ACK lysis solution and cells were washed two additional times with R10. Tissue-derived cells were used immediately for subsequent experiments.

### Study Cohort

Thirty-three chronically HCV-infected patients were recruited at the Department of Medicine II of the University Hospital Freiburg, Germany. Viral loads and transaminases of patients were determined as part of the clinical diagnostics at the University Hospital Freiburg. Patients with liver cirrhosis were excluded from the study. At the time of the study none of the patients chronically infected with HCV were receiving current antiviral treatment. Patient characteristics are listed in Table 1. Healthy controls were collected from the John Radcliffe Hospital, Oxford University Hospitals or recruited at the Department of Medicine II of the University of Freiburg Hospital.

**Table 1:** Characteristics of the hepatitis C virus (HCV) study cohort.

No.	Diagnosis	Sex	Age (years)	Viral load at analysis (IU/ml)	ALT (U/l)	Genotype
C01	Chronic HCV	F	45	3,329,040	151	1a
C02	Chronic HCV	M	62	nd	57	1a
C03	Chronic HCV	F	41	nd	32	1a
C04	Chronic HCV	F	53	nd	37	1b
C05	Chronic HCV	M	35	3,112,341	80	3a
C06	Chronic HCV	M	43	2,906,681	70	1a
C07	Chronic HCV	F	39	nd	30	1a
C08	Chronic HCV	M	43	nd	45	1a
C09	Chronic HCV	F	38	317,284	186	1a
C10	Chronic HCV	M	57	452,725	204	1a
C11	Chronic HCV	M	58	1,670,744	64	1b
C12	Chronic HCV	M	60	nd	86	3a
C13	Chronic HCV	M	57	nd	54	1b
C14	Chronic HCV	F	53	398,025	47	1b
C15	Chronic HCV	F	32	516,631	59	3a
C16	Chronic HCV	F	53	nd	36	3a
C17	Chronic HCV	F	20	1055,355	57	1b
C18	Chronic HCV	M	61	nd	92	1a
C19	Chronic HCV	F	39	3,394,892	61	1b
C20	Chronic HCV	M	35	8,336	206	1a
C21	Chronic HCV	F	51	2,897,088	70	1b
C22	Chronic HCV	M	42	nd	75	3a
C23	Chronic HCV	M	32	3,896,798	86	1b
C24	Chronic HCV	M	35	nd	189	3a
C25	Chronic HCV	F	32	nd	32	3a
C26	Chronic HCV	F	76	nd	21	1b
C27	Chronic HCV	F	49	26,121	32	3
C28	Chronic HCV	M	73	1,223,768	59	1b
C29	Chronic HCV	M	33	nd	104	1b
C30	Chronic HCV	M	52	499,162	120	3a
C31	Chronic HCV	M	48	nd	25	1b
C32	Chronic HCV	F	29	1,518,936	29	1a
C33	Chronic HCV	M	48	nd	26	2

### *In Vitro* Stimulation

*In vitro* stimulation was performed as previously described (2). Briefly, purified single cell suspensions of blood-, liver-, and duodenum-derived mononuclear cells were plated at 10^6^ cells per well of a 96-well U-bottom plate. IL-12 (R&D Systems), IL-15 (R&D Systems), IL-18 (R&D Systems), or IFN-β (Sigma) were added at a final concentration of 50 ng/ml. Cells were incubated for 16 h at 37°C, 5% CO_2_. After 16 h, Brefeldin A (5 µg/ml final concentration), and Monensin (2 µM final concentration) were added and incubation was carried out for an additional 4 h. After 20 h of total stimulation, intracellular cytokine staining was performed as described below. For PMA (phorbol 12-myristate 13-acetate) and ionomycin stimulation, Cell Activation Cocktail (BioLegend) was used per the manufacturer’s instructions in the presence of Brefeldin A and Monensin, and cells were incubated for 4 h at 37°C, 5% CO_2_.

### Flow Cytometry

For surface markers, cells were stained in a 50 µl volume of FACS buffer (PBS + 0.05% BSA + 1% penicillin/streptomycin) for 30 min at 4°C. Surface antibodies were: CD3 (clones OKT3 or UCHT1), Vδ1 (clone REA173), Vδ2 (clone B6), Vα7.2 (clone 3C10), CD161 (clone 191B8), IL-18Rα (clone H44 or 70625), CCR6 (clone G034E3), and fixable viability dye (ThermoFisher). Specifically for CCR6, staining was performed at 37°C for 30 min. Cells were washed once in FACS buffer and then standard surface staining was performed. For intracellular cytokine staining, surface staining was performed, as above, and then cells were fixed and permeabilized by 20 min incubation at 4°C in Cytofix/Cytoperm (BD Biosciences). Cells were washed twice in BD Perm/Wash Buffer (BD Biosciences). Intracellular staining for Granzyme B (clone GB11), IFN-γ (clone B27), and IL-17A (clone BL168) was performed in a 50 µl volume of BD Perm/Wash Buffer for 30 min at 4°C. For transcription factor staining, staining of surface markers was performed, as above, and then cells were fixed for 1 h at room temperature using FoxP3 Fixation Buffer (ThermoFisher). Cells were washed in FoxP3 kit Wash Buffer, and permeabilized for 1 h at room temperature in FoxP3 kit Wash Buffer. PLZF (clone R17-809) and RORγt (clone Q21-559) transcription factors were stained in a 50 µl volume of FoxP3 kit Wash Buffer for 1 h at room temperature. Following staining, cells were stored at 4°C until data acquisition. All antibodies were purchased from BD Biosciences, BioLegend, Miltenyi Biotec, or R&D Systems.

### Data Acquisition and Statistics

Data were acquired on a BD Fortessa flow cytometer or BD LSRII flow cytometer (BD Biosciences) and were analyzed using FlowJo version 9.9.5 (FlowJo, LLC). Statistical analyses were performed using Prism version 7 (GraphPad Software).

## Results

### Vδ2^+^ γδT Cells Can Be Activated by Cytokines in a TCR-Independent Manner

**Figure 1:**
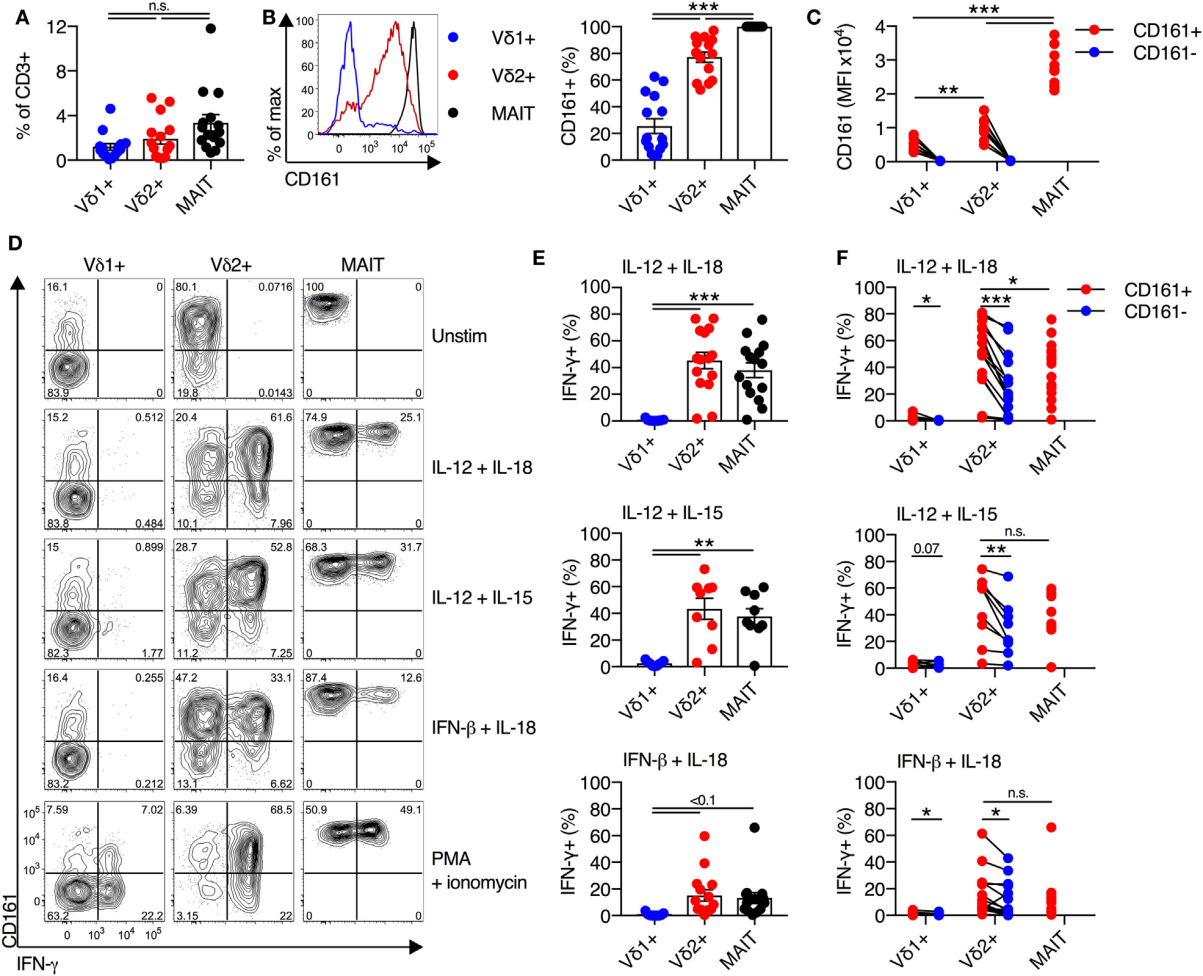
Vδ2^+^ γδT cells express CD161 and produce IFN-γ in response to cytokine stimulation. **(A)** Frequency of Vδ1^+^ γδT cells, Vδ2^+^ γδT cells, and mucosal-associated invariant T (MAIT) (Vα7.2^+^CD161^++^) cells in human blood. **(B)** Representative FACS plot and group averages for expression of CD161. **(C)** Mean fluorescence intensity (MFI) of CD161 expression on each subset. **(D–F)** Human peripheral blood mononuclear cells were stimulated for 20 h with the indicated cytokines (50 ng/ml). Brefeldin A and Monensin were added for the final 4 h. **(D)** Representative FACS plots of IFN-γ production. **(E)** Production of IFN-γ by each cell subset in response to the indicated cytokines. **(F)** Production of IFN-γ segregated by CD161 expression for each cell subset. Mean ± SEM. Symbols represent individual donors (*N* = 9–15), pooled from two individual experiments, or representative of two experiments **(C)**. ***P < 0.001; **P < 0.01; *P < 0.05. Repeated measures paired ANOVA with correction for multiple comparisons **(A,B,E)** and paired *T* test **(C,F)**.

We sought to determine if distinct CD161 expression is linked to different cytokine responsiveness of Vδ2^+^ and Vδ1^+^ subsets of γδT cells, as these are the two most prominent subsets of γδT cells in the blood. Vδ1^+^ γδT cells and Vδ2^+^ γδT cells were both abundant in blood (mean of 1.2 and 1.9% of all CD3^+^ T cells, respectively) (Figure 1A), with no significant difference in frequency compared to MAIT cells. Vδ2^+^ γδT cells predominantly expressed CD161 (77%), compared to only 26% of Vδ1^+^ γδT cells (*P* < 0.001; Figure 1B), in line with previous reports (15–17). There was significantly greater per-cell expression of CD161 [as measured by mean fluorescence intensity (MFI)] on the CD161^+^ Vδ2^+^ γδT cells compared to the CD161^+^ Vδ1^+^ γδT cell subset (*P* < 0.01; Figure 1C). When compared to the MAIT cell population, a smaller fraction of Vδ2^+^ γδT cells expressed CD161 and had reduced per-cell expression of CD161 (*P* < 0.001; Figures 1B,C). Thus, based on CD161 expression, Vδ2^+^ γδT cells appeared more similar to MAIT cells than the Vδ1^+^ γδT cell population.

We next sought to determine if the production of IFN-γ by CD161^+^ γδT cells in response to TCR-independent cytokine stimulation occurred in both the Vδ1^+^ and Vδ2^+^ γδT cell subsets. Combinatorial stimulation with IL-12 + IL-18, IL-12 + IL-15, or IFN-β + IL-18 for 20 h was performed (Figure 1D), as these cytokine combinations have been previously shown to potently activate MAIT cells (2, 3). In response to cytokine stimulation, a large fraction of Vδ2^+^ γδT cells and MAIT cells produced IFN-γ, while production of IFN-γ by Vδ1^+^ γδT cells was negligible (Figures 1D,E). For all three stimuli, equivalent responses by Vδ2^+^ γδT cells and MAIT cells were observed. IL-12 + IL-18 and IL-12 + IL-15 were the most potent stimuli (45% IFN-γ^+^ and 43% IFN-γ^+^ of Vδ2^+^ γδT cells, respectively), while IFN-β + IL-18 was the least stimulatory (15% IFN-γ^+^ of Vδ2^+^ γδT cells). Intriguingly, within the Vδ2^+^ γδT cell population, both CD161^+^ and CD161^−^ cells produced IFN-γ in response to all three stimuli (Figure 1F). However, a significantly larger fraction of CD161^+^ Vδ2^+^ γδT cells produced IFN-γ than the CD161^−^ subset (*P* < 0.05; Figure 1F), consistent with a previous report of increased cytokine-responsiveness in the CD161^+^ γδT cell population (8). For IL-12 + IL-18 stimulation, the CD161^+^ Vδ2^+^ γδT cells produced significantly more IFN-γ than MAIT cells (*P* < 0.05), but this difference was not observed following IL-12 + IL-15 or IFN-β + IL-18 stimulation. Despite very minor production of IFN-γ by Vδ1^+^ γδT cells in response to cytokine stimulation, there was a trend toward modestly increased IFN-γ production by the CD161^+^ fraction (Figure 1F). The fraction of Vδ1^+^ and Vδ2^+^ γδT cells that express CD161 did not change following cytokine stimulation (data not shown), suggesting that the IFN-γ producing CD161^−^ γδT cells do not represent downregulation of CD161 in response to stimulation. Overall, these data demonstrate that, akin to MAIT cells, the Vδ2^+^ γδT cell population has the inherent capacity to respond to cytokine stimulation. This corresponds to high expression of CD161 within this population, but cytokine responsiveness is an inherent trait seen in both the CD161^+^ and CD161^−^ subsets.

### Vδ2^+^ γδT Cells Express Key Characteristics of Innate-Like T Cells

**Figure 2:**
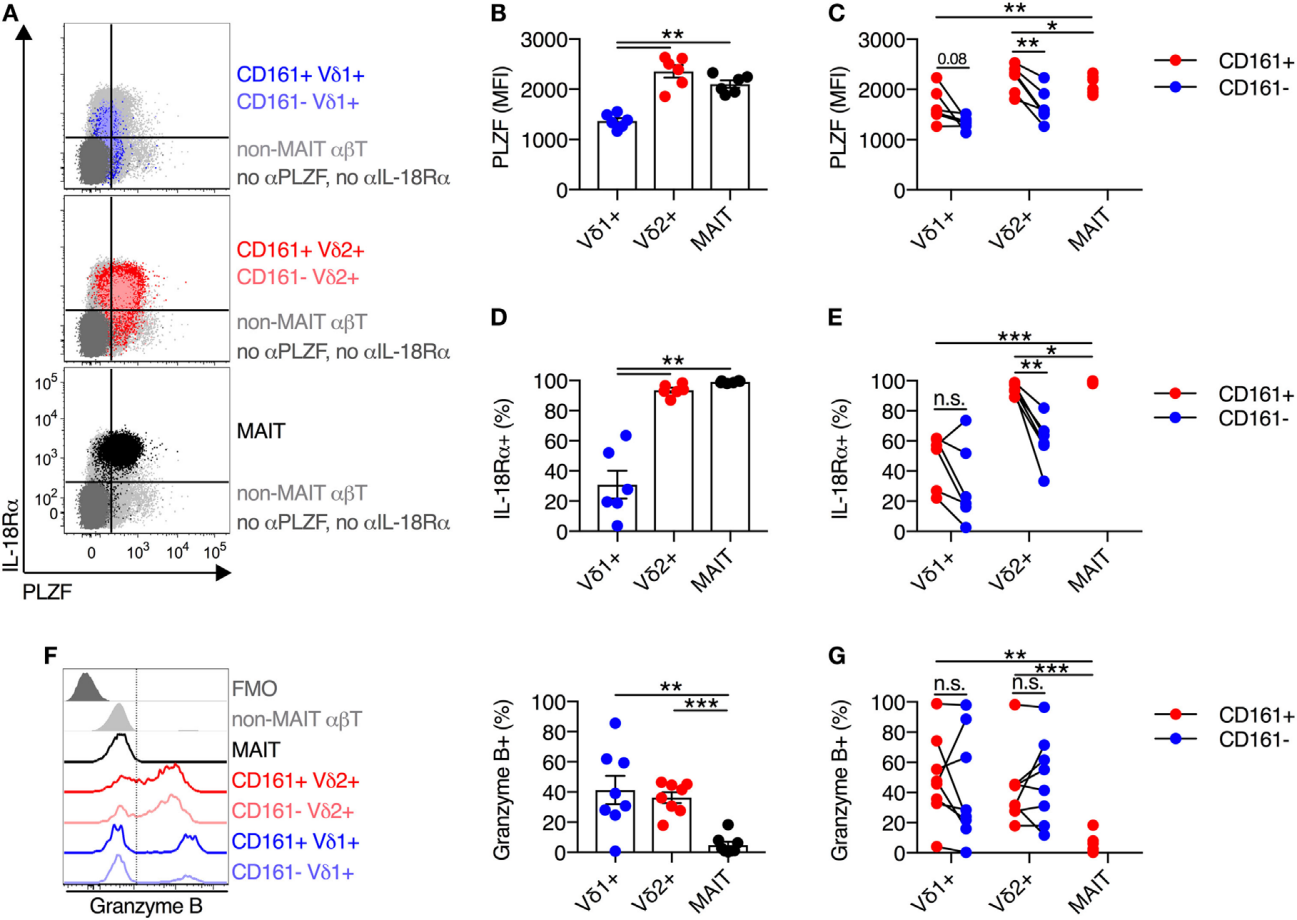
Vδ2^+^ γδT cells express high levels of IL-18Rα and PLZF. **(A)** Representative FACS plots for co-expression of PLZF and IL-18Rα on CD161^+^ or CD161^−^ Vδ1^+^ γδT cells and Vδ2^+^ γδT cells, and mucosal-associated invariant T (MAIT) (Vα7.2^+^ CD161^++^) cells. Non-MAIT αβT cells are included as a reference population, and cells not stained with αPLZF or αIL-18Rα antibodies are included as a control. **(B)** Group averages for expression of PLZF, measured by mean fluorescence intensity (MFI). **(C)** MFI of PLZF segregated by CD161 expression for each cell subset. **(D)** Group averages for expression of IL-18Rα, measured by percent expression. **(E)** Expression of IL-18Rα segregated by CD161 expression for each cell subset. **(F)** Representative FACS plot and group averages for expression of Granzyme B. Non-MAIT αβT cells are included as a reference population, and cells not stained with α Granzyme B antibodies (FMO) are included as a control. **(G)** Expression of Granzyme B segregated by CD161 expression for each cell subset. Mean ± SEM. Symbols represent individual donors (*N* = 6–8), representative of two independent experiments. ***P < 0.001; **P < 0.01; *P < 0.05. Repeated measures paired ANOVA with correction for multiple comparisons **(B,D,F)** and paired *T* test **(C,E,G)**.

Expression of the transcription factor PLZF is a key positive regulator of innate-like T cell function and development (18–20). Given the central role of PLZF in promoting innate-like T cell functionality, we sought to characterize PLZF expression in Vδ2^+^ γδT cells. Analogous to MAIT cells, Vδ2^+^ γδT cells expressed high levels of PLZF (Figures 2A,B), while PLZF expression was significantly lower on Vδ1^+^ γδT cells. When PLZF expression was assessed based on CD161 expression, both the CD161^+^ and CD161^−^ subsets of Vδ2^+^ γδT cells expressed high levels of PLZF, but expression was significantly higher in the CD161^+^ subset (*P* < 0.01; Figure 2C). Despite low expression of PLZF in the Vδ1^+^ γδT cell population, there was still a trend toward increased expression in the CD161^+^ subset (Figure 2C).

We additionally examined the expression of IL-18Rα, as expression of this receptor can be regulated by PLZF (21, 22), and expression is necessarily related to responsiveness to IL-18-mediated activation. The vast majority (>95%) of Vδ2^+^ γδT cells expressed IL-18Rα, while only 31% of Vδ1^+^ γδT cells expressed IL-18Rα (*P* < 0.01; Figures 2A,D). Co-expression of IL-18Rα and PLZF was observed on Vδ2^+^ γδT cells and MAIT cells (Figure 2A), as expected. Similar to PLZF, both the CD161^+^ and CD161^−^ subsets of Vδ2^+^ γδT cells expressed IL-18Rα at high levels, but again the CD161^+^ subset showed greater expression (*P* < 0.01; Figure 2E). Consistent with the inherent capacity of Vδ2^+^ γδT cells to respond to cytokine stimulation (Figure 1), the expression of PLZF and IL-18Rα is an inherent characteristic of these cells regardless of CD161 expression. However, CD161 expression correlated with increased expression of these molecules in line with the increased cytokine responsiveness, consistent with a previous report (8).

Intriguingly, in contrast to MAIT cells which exhibit negligible expression of Granzyme B in the steady state, approximately 40% of Vδ1^+^ γδT cells and Vδ2^+^ γδT cells expressed Granzyme B (*P* < 0.01; Figure 2F). Expression of Granzyme B did not vary between the CD161^+^ and CD161^−^ subsets of either γδT cell population (Figure 2G). Thus, while Vδ2^+^ γδT cells share phenotypic and transcriptional traits with MAIT cells, they do display differences with regard to baseline cytotoxic potential.

### Compared to MAIT Cells, Vδ2^+^ γδT Cells Have a Reduced Capacity to Perform Type 17 Effector Functions

**Figure 3:**
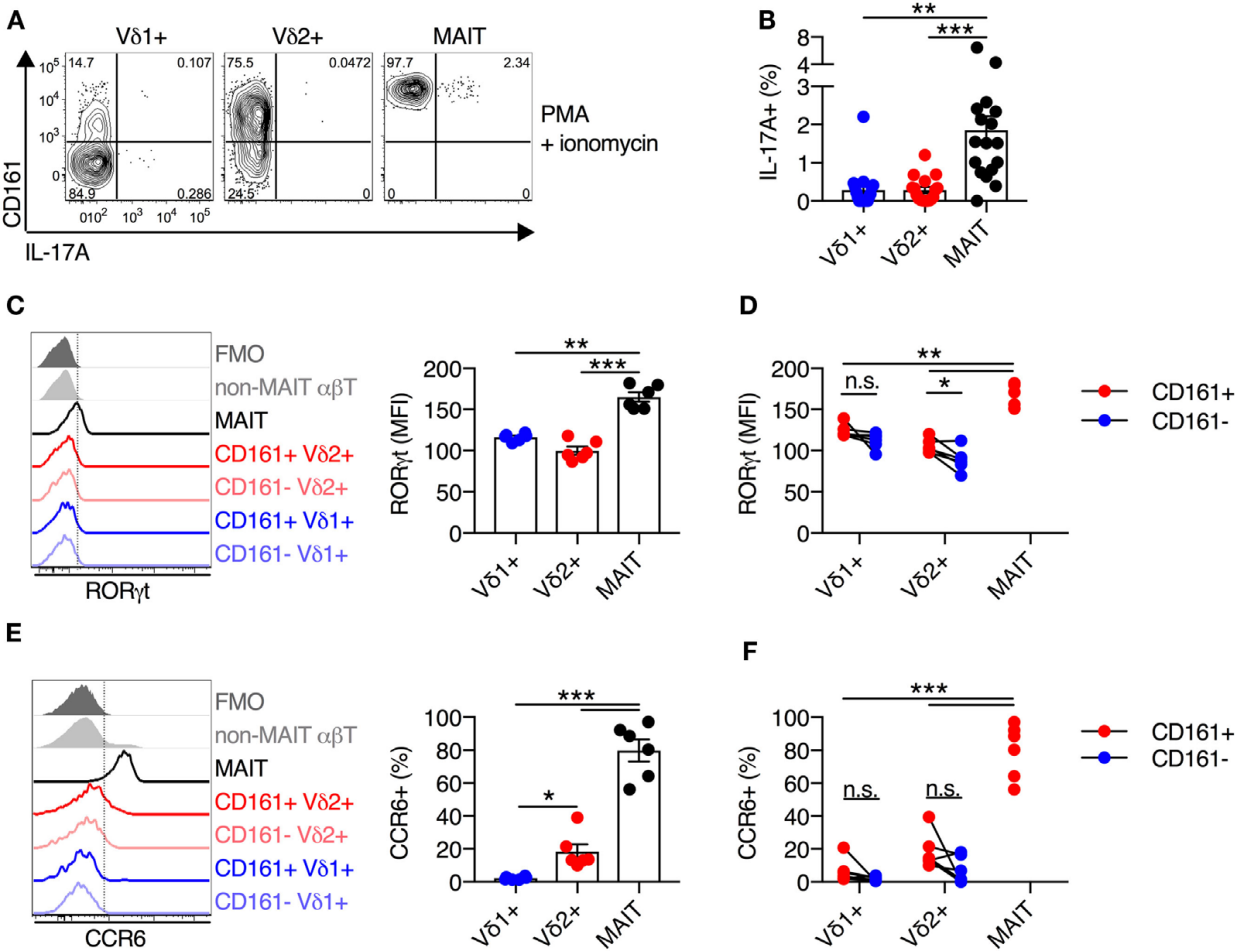
Blood Vδ2^+^ γδT cells display a reduced type 17 immune phenotype compared to mucosal-associated invariant T (MAIT) cells. **(A,B)** Human peripheral blood mononuclear cells were stimulated for 4 h with PMA and ionomycin, and Brefeldin A and Monensin were added at the time of stimulation. Representative FACS plots **(A)** and group averages **(B)** for production of IL-17A. **(C)** Representative FACS plot and group averages for expression of RORγt. Non-MAIT αβT cells are included as a reference population, and cells not stained with αRORγt antibodies (FMO) are included as a control. **(D)** Expression of RORγt segregated by CD161 expression for each cell subset. **(E)** Representative FACS plot and group averages for the percent expression of CCR6. Non-MAIT αβT cells are included as a reference population, and cells not stained with αCCR6 antibodies (FMO) are included as a control. **(F)** Percent expression of CCR6 segregated by CD161 expression for each cell subset. Mean ± SEM. Symbols represent individual donors (*N* = 6–9), representative of two independent experiments. ***P < 0.001; **P < 0.01; *P < 0.05. Repeated measures paired ANOVA with correction for multiple comparisons **(B,C,E)** and paired *T* test **(D,F)**.

Mucosal-associated invariant T cells have the capacity to execute type 17 effector functions, in addition to standard functionality of cytotoxic T lymphocytes (7, 23). Given the high degree of functional and phenotypic similarity observed thus far between Vδ2^+^ γδT cells and MAIT cells, we sought to determine if Vδ2^+^ γδT cells also had the capacity to produce IL-17A. As cytokine stimulation in the absence of TCR signaling does not induce IL-17A production by MAIT cells (data not shown), PMA + ionomycin stimulation was utilized. This stimulus led to IL-17A production by a small subset (mean of 2%) of MAIT cells (Figures 3A,B), in line with a previous report (23). However, IL-17A production by Vδ1^+^ γδT cells and Vδ2^+^ γδT cells was negligible (mean of 0.3% for both populations) and significantly lower than MAIT cell production (*P* < 0.01; Figures 3A,B).

Given the lack of IL-17A production by Vδ2^+^ γδT cells, we assessed the expression of the key transcriptional regulator of type 17 T cell functionality, RORγt. Consistent with the lack of IL-17A production by Vδ2^+^ and Vδ1^+^ γδT cells, both of these cell populations had significantly lower expression of RORγt as compared to MAIT cells (*P* < 0.01; Figure 3C). While, RORγt MFI was modestly increased within the CD161^+^ subset of Vδ2^+^ γδT cells, it did not approach levels seen in MAIT cells (*P* < 0.01; Figure 3D). In line with the low expression of RORγt, only a very small fraction of Vδ1^+^ and Vδ2^+^ γδT cells expressed CCR6, and expression was not increased on the CD161^+^ subsets (Figures 3E,F). While Vδ2^+^ γδT cells share innate-like functionality with MAIT cells, the two populations do not share the capacity to execute type 17 effector functions.

### Human Intestinal and Hepatic Vδ2^+^ γδT Cells Also Exhibit TCR-Independent Activation Potential

**Figure 4:**
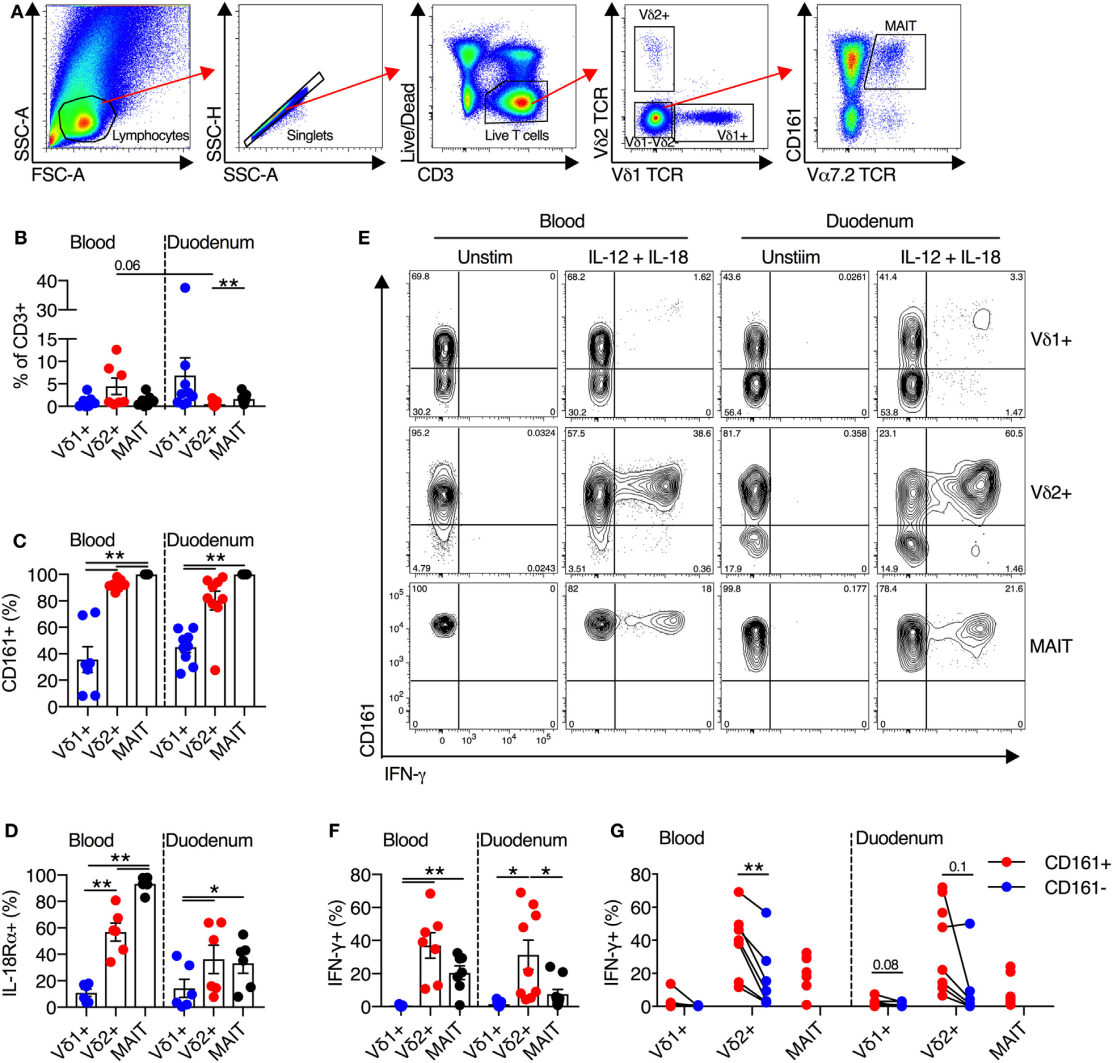
Human duodenal Vδ2^+^ γδT cells maintain innate-like phenotype and function. Intestinal mononuclear cells were isolated from biopsies of human duodenal tissue and matched blood. **(A)** Representative gating scheme for the identification of Vδ1^+^ γδT cells, Vδ2^+^ γδT cells, and mucosal-associated invariant T (MAIT) cells. **(B)** Frequency of Vδ1^+^ γδT cells, Vδ2^+^ γδT cells, and MAIT cells as a fraction of all CD3^+^ T cells in duodenum and matched blood. **(C)** Expression of CD161 on blood and matched duodenal Vδ1^+^ γδT cells, Vδ2^+^ γδT cells, and MAIT cells. **(D)** Expression of IL-18Rα on blood and matched duodenal Vδ1^+^ γδT cells, Vδ2^+^ γδT cells, and MAIT cells. **(E–G)** Blood and duodenal mononuclear cells were stimulated for 20 h with IL-12 and IL-18 (50 ng/ml). Brefeldin A and Monensin were added for the final 4 h. **(E)** Representative FACS plots of IFN-γ production by Vδ1^+^ γδT cells, Vδ2^+^ γδT cells, and MAIT cells. **(F)** Production of IFN-γ by each cell subset. **(G)** Production of IFN-γ segregated by CD161 expression for each cell subset. Note: three individuals had 100% CD161^+^ in the Vδ2^+^ γδT cell population, hence the lack of a paired comparator population. Mean ± SEM. Symbols represent individual donors (*N* = 8–9), pooled from four independent experiments. **P < 0.01; *P < 0.05. Repeated measures paired ANOVA with correction for multiple comparisons **(B–D,F)** and paired *T* test **(G)**.

Mucosal-associated invariant T cells are present at a high frequency in human intestinal and liver tissues (24–27). Thus, we next sought to determine the frequency of Vδ2^+^ γδT cells in these tissues, and if these cells maintained their innate-like capacity within tissues. We first examined the frequency and functionality of cells extracted from human duodenal biopsies (Figure 4A). Vδ2^+^ γδT cells comprised on average 0.5% of all duodenal CD3^+^ T cells, and this represented a trend toward reduced frequency compared to blood (*P* = 0.06; Figure 4B). No significant differences in frequency of Vδ1^+^ γδT cells and MAIT cells between blood and duodenum were observed. Consistent with blood, >80% of Vδ2^+^ γδT cells in the duodenum expressed CD161, while expression was significantly lower in the Vδ1^+^ γδT cell population (*P* < 0.01; Figure 4C). Both MAIT cells and Vδ2^+^ γδT cells from the duodenum had greater expression of IL-18Rα than the Vδ1^+^ γδT cell population (*P* < 0.05; Figure 4D). We next stimulated duodenum-derived mononuclear cells with IL-12 + IL-18. Analogous to blood-derived cells, the Vδ2^+^ γδT cell population responded robustly to this stimulus by producing IFN-γ (*P* = 0.7; Figures 4E,F), while the Vδ1^+^ γδT cell population was minimally responsive. The expression of CD161 on either the Vδ2^+^ or Vδ1^+^ γδT cells did not significantly affect production of IFN-γ in response to IL-12 + IL-18 stimulation, although a trend was observed (*P* ≤ 0.1; Figure 4G). Akin to blood, Vδ2^+^ γδT cells from the duodenum were activated by IL-12 + IL-18 stimulation regardless of expression of CD161.

**Figure 5:**
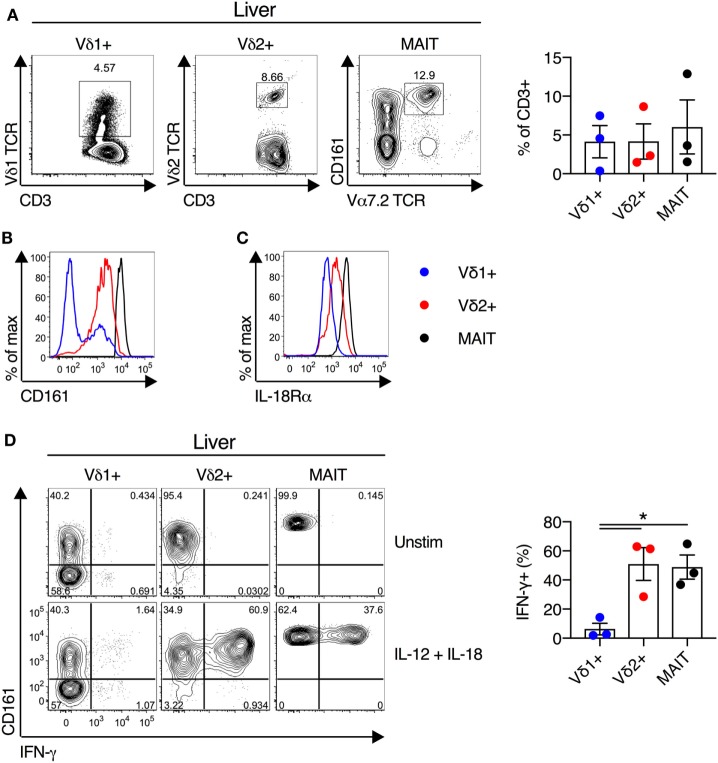
Human liver Vδ2^+^ γδT cells maintain innate-like phenotype and function. Liver mononuclear cells were isolated from uninvolved tissue of colorectal cancer metastases or hepatocellular carcinoma patients. **(A)** Representative FACS plots and frequency of Vδ1^+^ γδT cells, Vδ2^+^ γδT cells, and mucosal-associated invariant T (MAIT) cells as a fraction of all liver CD3^+^ T cells. **(B)** Expression of CD161 on liver Vδ1^+^ γδT cells, Vδ2^+^ γδT cells, and MAIT cells. **(C)** Expression of IL-18Rα on liver Vδ1^+^ γδT cells, Vδ2^+^ γδT cells, and MAIT cells. **(D)** Liver mononuclear cells were stimulated for 20 h with IL-12 and IL-18 (50 ng/ml). Brefeldin A and Monensin were added for the final 4 h. Representative FACS plots and group summary of IFN-γ production by each cell subset in response to cytokine stimulation. Mean ± SEM. Symbols represent individual donors (*N* = 3), pooled from three independent experiments. *P < 0.05. Paired *T* test.

We next examined the functionality of liver-derived γδT cells. Vδ1^+^ γδT cells, Vδ2^+^ γδT cells, and MAIT cells were abundant in human liver (mean of 4, 4, and 6% of CD3^+^ T cells for Vδ1^+^ γδT cells, Vδ2^+^ γδT cells, and MAIT cells, respectively; Figure 5A). In all other respects liver-derived Vδ2^+^ γδT cells appeared functionally and phenotypically equivalent to blood- and duodenum-derived cells. Liver Vδ2^+^ γδT cells expressed high levels of CD161 and IL-18Rα (Figures 5B,C). Furthermore, IL-12 + IL-18 stimulation resulted in robust IFN-γ production by Vδ2^+^ γδT cells, comparable to MAIT cells, and these responses were significantly larger than seen in Vδ1^+^ γδT cells (*P* < 0.05; Figure 5D). In sum, Vδ2^+^ γδT cells derived from intestinal and liver tissues maintain their phenotype and responsiveness to cytokine stimulation.

### Vδ2^+^ γδT Cell Frequency and Innate-Like Functionality Is Maintained During Chronic HCV Infection

**Figure 6:**
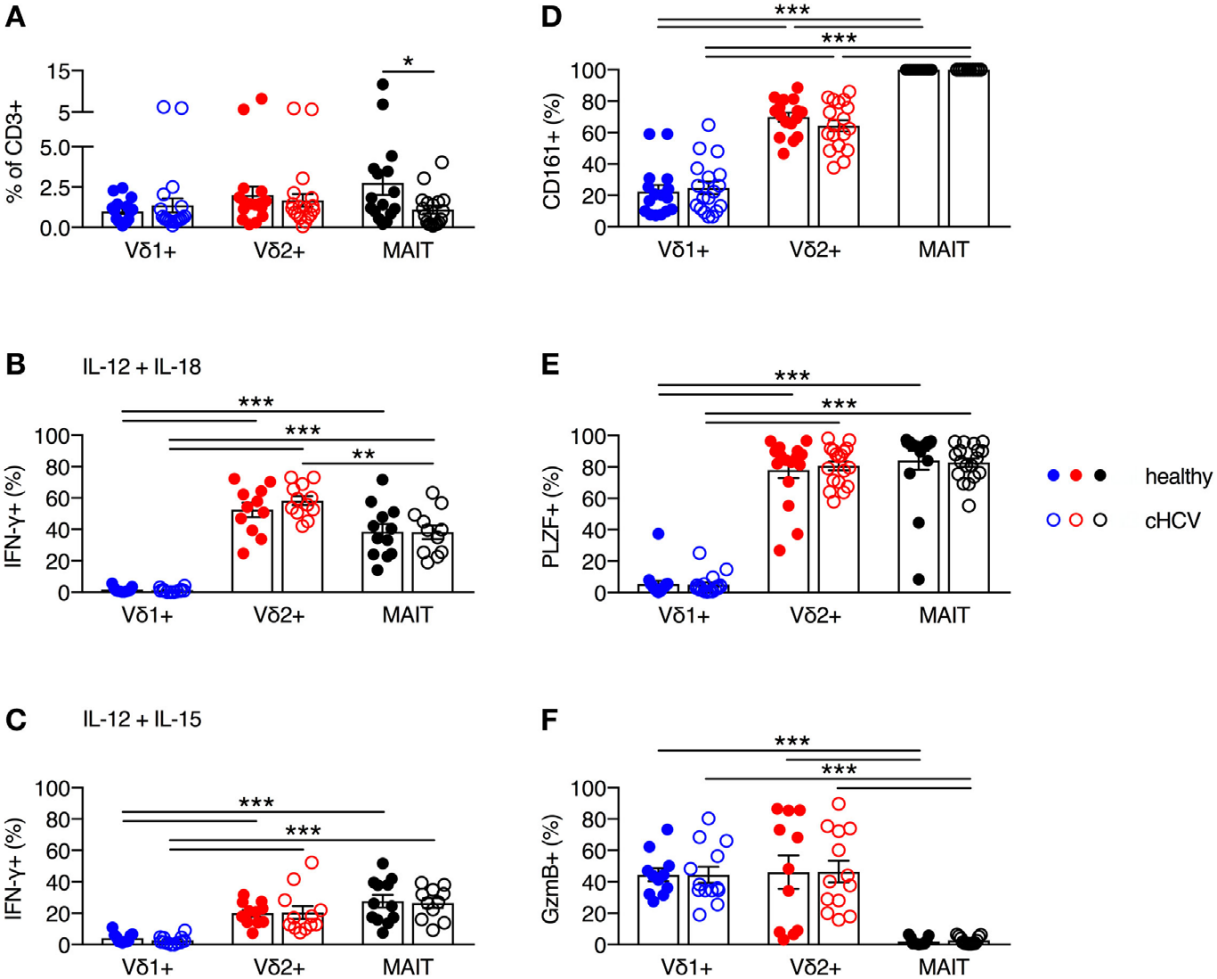
Peripheral blood Vδ2^+^ γδT cell frequency and cytokine-dependent function is maintained during chronic hepatitis C virus (cHCV) infection. **(A)** Frequency of Vδ1^+^ γδT cells, Vδ2^+^ γδT cells, and mucosal-associated invariant T (MAIT) cells as a fraction of all CD3^+^ T cells in the blood of healthy donors and patients with cHCV. **(B,C)** Peripheral blood mononuclear cells were stimulated for 20 h with IL-12 and IL-18 **(B)**, or IL-12 and IL-15 **(C)** (50 ng/ml). Brefeldin A and Monensin were added for the final 4 h. Production of IFN-γ by each cell subset from healthy donors and HCV patients. **(D–F)** Expression of CD161 **(D)**, PLZF **(E)**, and Granzyme B **(F)** on Vδ1^+^ γδT cells, Vδ2^+^ γδT cells, and MAIT cells. Mean ± SEM. Symbols represent individual donors (*N* = 11–33). ***P < 0.001; **P < 0.01; *P < 0.05. **(A)** *T* test and **(B–F)** two-way ANOVA with Tukey’s multiple comparison test.

It has been previously reported that MAIT cells can be activated *in vitro* and *in vivo* by HCV *via* cytokines, and that MAIT cells can inhibit HCV replication *in vitro* (3). Furthermore, chronic HCV infection has been shown to reduce the frequency of circulating and liver MAIT cells (3, 28–31), but no impairment in IFN-γ production in response to cytokine-mediated activation has been observed (28–31). Although several studies have examined alterations in frequency and functionality of γδT cells during chronic HCV infection (32–34), the phenotype and functionality of Vδ2^+^ γδT cells has not been examined in detail. Thus, we sought to determine the impact of chronic HCV infection on the frequency and functionality of Vδ2^+^ γδT cells. We utilized a cohort of chronically HCV-infected individuals (Table 1) and matched controls. Chronic HCV infection significantly reduced the frequency of circulating MAIT cells (*P* < 0.05; Figure 6A). By contrast, the frequencies of Vδ1^+^ γδT cells and Vδ2^+^ γδT cells were unaffected by chronic HCV infection (Figure 6A). Vδ2^+^ γδT cells from chronically HCV-infected individuals also displayed unimpaired production of IFN-γ in response to IL-12 + IL-18 or IL-12 + IL-15 stimulation (Figures 6B,C). In healthy controls, Vδ2^+^ γδT cells and MAIT cells responded equivalently to IL-12 + IL-18 stimulation (Figure 6B). By contrast, chronically HCV-infected patients showed increased responsiveness of Vδ2^+^ γδT cells to IL-12 + IL-18 stimulation compared to MAIT cells (Figure 6B). However, this difference was not seen following IL-12 + IL-15 stimulation (Figure 6C). Consistent with the maintained functionality, Vδ2^+^ γδT cells from chronically HCV-infected individuals expressed high levels of CD161, PLZF, and Granzyme B, equivalent to levels seen in Vδ2^+^ γδT cells from healthy controls (Figures 6D–F). Vδ1^+^ γδT cells, Vδ2^+^ γδT cells, and MAIT cells from chronically HCV-infected patients maintained the differences in expression of CD161, PLZF, and Granzyme B that was observed in healthy controls. In sum, chronic HCV infection does not appear to impact on the frequency, phenotype, or innate-like functionality of Vδ2^+^ γδT cells, thus highlighting a difference between Vδ2^+^ γδT cells and MAIT cells.

## Discussion

In the current study, we demonstrate that Vδ2^+^ γδT cells are a major population of innate-like, cytokine-responsive T cells, which mirror MAIT cells with regard to frequency, phenotype, and innate-like functionality. In line with previous studies (15–17), we have determined that Vδ2^+^ γδT cells are the major population of CD161^+^ γδT cells, and that the CD161^+^ subset displays enhanced innate-like phenotype and functionality. Thus, we conclude that the prior report that CD161^+^ γδT cells are a cytokine-responsive population (8), actually identifies the Vδ2^+^ γδT cell population. Collectively, this study demonstrates the highly similar biology of human MAIT and Vδ2^+^ γδT cells with regard to TCR-independent, cytokine-driven activation. These data highlight the importance of examining both cell populations when considering cytokine-mediated activation of lymphocyte populations.

One area of particular interest with regard to cytokine-mediated activation is in the context of viral infections. While neither MAIT cells, whose TCRs recognize riboflavin-based metabolites in the context of MR1 (35), nor Vδ2^+^ γδT cells, whose TCRs recognize phospho-antigens through Butyrophilin 3A1 (36, 37), can directly sense virus-derived antigens, both populations can be activated by viral infections *in vitro* (3, 6, 13). These studies also reported *in vivo* activation of MAIT cells in patients following influenza, dengue, or HCV infection (3, 6). Analysis of *in vivo* Vδ2^+^ γδT cell activation was not performed. For all viral infections studied, both Type I IFNs and IL-18 were critical for the activation of these cell populations, thus highlighting the biologic relevance of this cytokine-mediated activation pathway. Using the most reductionist system of recombinant cytokines in the absence of ongoing viral infection, our data confirm the observation that cytokines can activate Vδ2^+^ γδT cells to carry out effector functions. While the exact impact of virus-mediated activation of MAIT cells or Vδ2^+^ γδT cells on disease outcomes remains to be determined, our data illustrate that both populations are equally responsive to this mechanism of activation. Thus, it is important for future studies examining the role of innate-like T cells in human viral infections to consider both MAIT and Vδ2^+^ γδT cells.

Toward this point, the impact of chronic HCV infection on MAIT cell frequency and function has been examined extensively, but the same is not true for the Vδ2^+^ γδT cell population. MAIT cell frequencies decline in both the circulation and in the liver during HCV infection (3, 28–31). We observed no significant reduction in the frequency of circulating Vδ2^+^ γδT cells in chronically HCV-infected patients, in contrast to a previous report (32). The reasons for the differences in reported frequency of Vδ2^+^ γδT cells between cohorts of HCV-infected patients remain unclear. However, our cohort had lower serum ALT levels (71.35 ± 11.49; Table 1) compared to the prior study (165.5 ± 16), which suggests that degree of tissue damage may impact on the frequency of Vδ2^+^ γδT cells during chronic HCV infection. However, it is clear that further investigation is required to better elucidate any interplay between HCV infection and Vδ2^+^ γδT cell frequency especially in the liver, the site of infection. We found that circulating Vδ2^+^ γδT cells from HCV-infected patients displayed an unimpaired capacity to produce IFN-γ in response to cytokine stimulation, and consistent with this, these cells maintained expression of PLZF. MAIT cells from chronically HCV-infected individuals, despite reduced frequencies, also maintained the capacity for cytokine-induced production of IFN-γ (Figure 6), consistent with prior reports (28–31). Thus, it appears that innate-like functionality can be maintained in the setting of chronic viral infection, despite reduced TCR-mediated functionality (28, 30). This characteristic makes both MAIT cells and Vδ2^+^ γδT cells attractive therapeutic targets, as both cells have been shown to be capable of inhibiting HCV replicons *in vitro* (3, 38).

While Vδ2^+^ γδT cells and MAIT cell frequencies are differentially affected by chronic HCV infection, we report many shared characteristics between these two populations with regard to tissue distribution, innate-like phenotype, and responsiveness to cytokine stimulation. However, there are two key fundamental biologic traits where the functionalities of these cell populations diverge. First, Vδ2^+^ γδT cells constitutively express Granzyme B (Figure 2) (39), but with large individual-to-individual variation in steady-state expression (40). By contrast, at rest, MAIT cells do not express Granzyme B (Figure 2) and instead must undergo licensing before expression occurs (41). While both populations can ultimately secrete fully loaded cytotoxic granules, these differences in steady-state composition may have important implications for the kinetics of the cytotoxic response from each population. Second, MAIT cells display a mixed Tc1/Th17 phenotype with the ability to produce IFN-γ and cytotoxic granules, while also producing IL-17A and IL-22 (42). This correlates with high expression of the Th17-driving transcription factor RORγt and Th17-associated markers such as CCR6 and IL-23R (7, 23). By contrast, in this comparative study, we could not detect type 17 immune functionality for Vδ2^+^ γδT cells (Figure 3), and this corresponded to low expression of RORγt and absence of CCR6. These data suggest that Vδ2^+^ γδT cells are minor innate-like type 17 effector cells compared to MAIT cells.

Detailed transcriptional comparisons of MAIT and Vδ2^+^ γδT cells will enhance our understanding of the regulatory networks and pathways involved in regulating innate-like T cells. It appears that the difference in type 17 functionality of Vδ2^+^ γδT cells and MAIT cells represents an example of different regulatory networks associated with the core innate-like T lymphocyte transcriptional program. Both innate-like T cell populations express PLZF to similar levels, but differ with regard to type 17 effector functionality. Intriguingly, it has been reported that PLZF regulates expression of RORγt and thus type 17 effector functionality in a context-dependent manner. In particular, it has been reported on the one hand that PLZF can downregulate RORγt (43), and on the other hand, it controls IL-17A-producing cord blood-derived γδT cell maturation (44). Thus, a better understanding of how PLZF might be differentially regulating gene expression in Vδ2^+^ γδT cells and MAIT cells is of considerable interest.

Detailed transcriptional comparisons of MAIT and Vδ2^+^ γδT cells will also enhance our understanding of the true core factors and pathways involved in innate-like functionality of human T cells. We have previously reported such a “cytokine-responsive” transcriptional signature derived from human MAIT cells (8). Expression of CD161 was defined as a core marker of this transcriptional signature, but we now demonstrate that both CD161^+^ and CD161^−^ Vδ2^+^ γδT cells robustly produce IFN-γ in response to cytokine stimulation, albeit with enhanced responses in the CD161^+^ subset. With our increased understanding of Vδ2^+^ γδT cell biology, hopefully this transcriptional signature can be further refined to identify core genes necessary for cytokine-dependent, TCR-independent activation of human T cells.

In conclusion, human Vδ2^+^ γδT cells and MAIT cells represent two innate-like T cell populations with overlapping tissue distribution and responsiveness to cytokine-mediated activation. However, MAIT cells and Vδ2^+^ γδT cells differ in their ability to execute type 17 effector functions and baseline cytotoxic granule composition. As a case study, we demonstrate that circulating Vδ2^+^ γδT cells are maintained in frequency and functionality during chronic HCV infection, a disease where MAIT cell numbers decline (3, 28–31). In sum, this study not only links CD161 expression to the proficient cytokine responsiveness of Vδ2^+^ γδT cells but also sheds light on the overlapping nature, accompanied by unique features, of innate-like T cells in the circulation and within tissues exemplified by Vδ2^+^ γδT cells and MAIT cells. In particular, the differential impact of chronic HCV infection on these populations reveals the necessity for both innate-like T cell subsets to be considered in the complex immune interplay of a clinically relevant viral infection.

## Ethics Statement

Written informed consent was received from all subjects in accordance with the Declaration of Helsinki. Duodenal tissue, liver tissue, and matched blood samples were collected under a specific project approved under the study, “Gastrointestinal Illness in Oxford: prospective cohort for outcomes, treatment, predictors and biobanking” which in turn was approved by the relevant local research ethics committee (Ref: 11/YH/0020). Furthermore, blood samples from chronically HCV-infected patients or healthy blood donors and liver tissue were collected through the “Hepatologische Biobank des Universitaetsklinikums Freiburg” (HBUF; 474/14) under the specific project Ref: 275/15 approved by the local ethics committee. Peripheral bloods were also acquired from anonymized healthy blood donors through the NHS Blood and Transplant Service.

## Author Contributions

NP, MF, BB, and AS performed experiments. KW and LG prepared and provided samples. NP, MF, BB, AS, BW, RT, MH, and PK designed the experiments. All authors contributed to the writing and editing of the manuscript.

## Conflict of Interest Statement

The authors declare that the research was conducted in the absence of any commercial or financial relationships that could be construed as a potential conflict of interest.
